# Validation of a field-friendly faeces drying and storage method for quantifying faecal glucocorticoid metabolites in African elephants (Loxodonta africana) opens up new perspectives for conservationists

**DOI:** 10.1093/conphys/coad053

**Published:** 2023-07-31

**Authors:** Laura Lacomme, Chloé Guerbois, Hervé Fritz, André Ganswindt, Benjamin Rey

**Affiliations:** REHABS International Research Laboratory, French National Centre for Scientific Research (CNRS), University of Lyon 1, Nelson Mandela University, Madiba Drive, George 6529, South Africa; Mammal Research Institute (MRI), Department of Zoology and Entomology, Faculty of Natural and Agricultural Sciences, University of Pretoria, Hatfield Campus, Pretoria 0028, South Africa; Biometry and Evolutionary Biology laboratory (LBBE), French National Centre for Scientific Research (CNRS),Unit 5558, University of Lyon 1, 43 Bd du 11 novembre 1918, 69622 Villeurbanne cedex, France; Sustainability Research Unit, Nelson Mandela University, George Campus, Madiba Drive, George 6529, South Africa; Sustainability Research Unit, Nelson Mandela University, George Campus, Madiba Drive, George 6529, South Africa; Sustainability Research Unit, Nelson Mandela University, George Campus, Madiba Drive, George 6529, South Africa; Mammal Research Institute (MRI), Department of Zoology and Entomology, Faculty of Natural and Agricultural Sciences, University of Pretoria, Hatfield Campus, Pretoria 0028, South Africa; Biometry and Evolutionary Biology laboratory (LBBE), French National Centre for Scientific Research (CNRS),Unit 5558, University of Lyon 1, 43 Bd du 11 novembre 1918, 69622 Villeurbanne cedex, France

**Keywords:** Conservation, faeces, non-invasive method, steroids, stress hormone, wildlife welfare

## Abstract

Faecal glucocorticoid metabolites (fGCMs) are a relevant means of non-invasively assessing adrenocortical activity and thus, a key physiological stress response in wildlife populations. However, the widespread use of fGCMs as a stress-related biomarker in conservation biology is often hampered by the logistical challenge of storing collected faecal material frozen until it reaches the laboratory for analysis. Although alternative approaches to minimize potential alteration of fGCM composition post-defecation have been recently identified, there is to our knowledge, no satisfactory alternative method established for the preservation of elephant dung. In this study, we validated a field-friendly protocol for dehydrating African elephant faeces samples using a food dehydrator with desiccant and investigated the stability of fGCM concentrations in the dehydrated faeces when stored at ambient temperature. We collected 40 faecal samples from African elephants and compared fGCM concentrations of freeze-dried and dehydrated sample sub-sets. Samples dried in the field showed a slight but significant overall −6% reduction in fGCM concentration compared with frozen control samples. However, fGCM concentrations following field dehydration protocol match those of control samples with high accuracy, as evidenced by the low bias and strong coefficient of determination between the two approaches (R^2^ = 0.88). In addition, over nearly 2 months, storage time at ambient temperature of the dehydrated samples had no effect on the fGCM concentrations compared with those measured in the control samples (F-statistic = 1.82; *P* = 0.18). Dehydrating the samples in the field thus provides an easy and cost-effective alternative to freezing, especially when working in remote areas with unstable electrical supply. Our results encourage the widespread use of fGCMs by conservationists as non-invasive means of steroid monitoring of African elephants in the current context of a general increase in wildlife welfare research. Future studies are needed to extend the use of this protocol to other species and to other steroid classes.

## Teaser

Faecal glucocorticoid metabolites (fGCMs) provide relevant information on wildlife welfare but remain underapplied in conservation due to sample preservation issues. We validated a field-friendly drying and storage method for preserving African elephant faeces, addressing logistical challenges of fGCM quantification, while anticipating the growing interest in considering welfare in conservation management.

## Introduction

One of the major coping mechanisms to environmental or anthropogenic perturbations (e.g. lack of food, movement restraints, social disruption, human disturbance, translocation) is the stress response (Wingfield *et al.,* 1998), being defined as a suite of behavioural, physiological and neuroendocrine responses whose aim to neutralize the effect of stressors (Cannon, 1929). These days, markers of physiological stress as an indicator of wildlife disturbance and welfare are among the most popular tools in conservation biology (Busch and Hayward, 2009; Sheriff *et al.,* 2011). Among them, the quantification of faecal glucocorticoid metabolites (fGCMs) has received considerable attention because it has great potential as a non-invasive diagnostic of endocrine functions and as a decision aid for conservationists (Ganswindt *et al.,* 2012; Dantzer *et al.,* 2014; Palme, 2019).

In African and Asian elephants, fGCM concentrations vary according to environmental conditions, individual (e.g. body condition, behaviour, dominance rank, reproductive status) and group characteristics (e.g. group size and population demography) (Foley *et al.,* 2001; Ganswindt *et al.,* 2005, 2010; Viljoen *et al.,* 2008; Mumby *et al.,* 2015; Pokharel *et al.,* 2017; Garai *et al.,* 2022). Anthropogenic factors, such as human–production habitat, elephant working conditions and tourism density can also affect fGCM concentrations (Millspaugh *et al.,* 2007; Pokharel *et al.,* 2018; Kumar *et al.,* 2019; Grotto *et al.,* 2020; Szott *et al.,* 2020). As is the case in many species, measures of fGCMs are now being integrated as objective measures of welfare in African elephants (Garai *et al.,* 2022).

However, a barrier to the popularization and widespread use of fGCM monitoring among conservationists lies in logistic limitations to achieve restrictive sample collection and storage protocols in remote areas (Madliger *et al.,* 2018). To avoid potential alteration of fGCM composition post-defecation, the widely used standard procedure consists of collecting the faeces sample in a quick and defined period after deposition and keep it frozen at −20°C until steroid extraction and analysis (Sheriff *et al.,* 2011). The strict maintenance of a cold chain between sample collection and analysis in the laboratory is particularly important (Möstl *et al.,* 2005). The composition of fGCMs cannot only be altered by freezing and thawing cycles (Pappano *et al.,* 2010; Gholib *et al.,* 2017), but also by keeping samples at low above-zero temperature (Carbillet *et al.,* 2023), which poses serious logistical challenges in remote areas and hot climates. This has stimulated research into faeces preservation treatments other than freezing for field application. To be widely applicable in the field, the sample collection, pre-treatment and storage protocols must be easy to implement and low energy consuming, while ensuring biologically meaningful fGCMs values and that the signal is stable and repeatable. For example, the use of organic solvents to preserve faeces in the field has proved effective in some species (e.g. in black rhinos (Edwards *et al.,* 2014) and in primates (Khan *et al.,* 2002; Pappano *et al.,* 2010; Shutt *et al.,* 2012; Gholib *et al.,* 2014, 2018; Nugraha *et al.,* 2017)), but with different preservation duration among studies (from a few hours to several weeks). More recently, dehydration of faeces with silica beads has proved effective in preserving fGCMs in horses (Krueger *et al.,* 2019) and African lions (Fowler and Santymire, 2022). However, faeces preservation protocols need to be properly validated for each new species (Palme, 2019), so there is no evidence that any of these protocols are suitable for elephants.

In African elephants, alternatives to freezing protocols for faecal steroid metabolite preservation have been tested on a single captive elephant, including drying in an oven, or with silica gel, or immersing the samples in 90% ethanol, but the fGCM concentrations differed greatly in a time-dependent manner from those in samples preserved at −20°C (Hunt and Wasser, 2003). Simple drying protocols consisting of passive exposure of elephant dung to sunlight or shade also resulted in a drastic loss of fGCM concentration (Webber *et al.,* 2018), which may be attributed to the long drying time that allows for the alteration of the immunoreactive steroid metabolite composition in faeces presumably by bacterial enzymes (Bokkenheuser and Winter, 1980; Möstl *et al.,* 1999).

Very recently, a dehydration technique using a low-power supplied food dehydrator has been tested on African wild dog faeces and has shown encouraging results on the integrity of fGCMs (Postiglione *et al.,* 2022). Compared with other alternative drying processes, such as exposure to sunlight and the solar oven, this protocol fastens the removal of moisture from the faeces by combining heating with the circulation of a constant air flow. Therefore, the combined use of a food dehydrator and silica beads as a desiccant to trap water in the air inside the food dehydrator would further accelerate the drying process and prevent degradation of fGCMs by bacterial enzymes present in the faeces (Palme, 2019). Whether this protocol is relevant for African elephant dung remains an open question. The question also arises whether fGCMs in field-dehydrated samples are stable when preserved at ambient temperature. Hence, the aim of our study was i) to validate the method for dehydrating African elephant faeces using a food dehydrator and desiccant for subsequent measurement of fGCM concentrations and ii) to test the stability of field-dehydrated samples at ambient temperature over time.

## Material and Methods

All the procedures in this study received approval from the Nelson Mandela University Animal Research Ethic Committee (ethical clearance number A21-SCI-NRM-002).

### Samples collection

We collected faecal samples from 40 free-ranging African elephants in seven reserves of the North West, Limpopo and Kwa-Zulu Natal Provinces of South Africa from mid-August to late September 2021. All elephants were adults and included 15 males, 5 females and 20 unidentified. Elephants were tracked from a distance and all samples were collected within 1 hour after defecation. For each individual, 50 g of faecal material was sampled from the middle of the bolus to avoid cross-contamination with urine or debris (Ganswindt *et al.,* 2002). Wearing disposable gloves, the samples were kneaded thoroughly to ensure uniform distribution of hormones, and then split in two. One sub-sample was sealed in a plastic bag for subsequent freezing at −20°C and served as control. The second sub-sample was placed in a folded paper bag for subsequent dehydration protocol (Figure 2). In the field, samples were placed on ice packs in a cooler box before returning to the camp no more than 8 hours after collection. The samples contained in paper bags were carefully deposited in the cooler box so as not to come into direct contact with the ice packs, which would have wetted them.

### Dehydration and storage of faecal samples in the field

**Figure 1:**
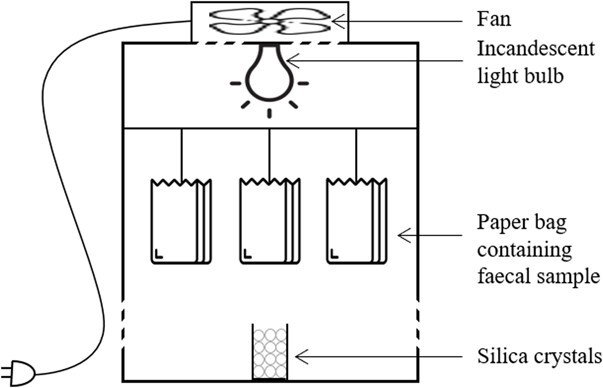
Diagram of the dehydrator assembly. The dehydration process was ensured by the heat released from the B22 incandescent bulb (60 W) and the constant air flow produced by the fan. We added 20 g of desiccant silica crystals in a small plastic container and we deposited the container open in the bottom of the dehydrator to hasten humidity absorption and optimize the drying process. Each paper bag containing the faecal sample of a single individual was widely opened and hooked onto a support bar with wire, hanging in the dehydrator.

Dehydration of faecal samples was performed in a food dehydrator (model BK002 Mellerware, Johannesburg, South Africa) for 24 h (Figure 1). The dehydration process was ensured by heating with a B22 incandescent bulb (60 W) and a constant air flow. We added 20 g of desiccant silica crystals in a small plastic container and we deposited the container open in the bottom of the dehydrator to hasten humidity absorption and optimize the drying process. Each paper bag containing the faecal sample of a single individual was widely opened and hooked with wire to hang in the dehydrator. The dehydrator contained from one to six samples at a time. After 24 h of drying, the samples were transferred, sealed in plastic containers and maintained at ambient temperature (15–25°C) until assayed. The average time from dehydration to hormone assay was 34 ± 12 SD days and ranged from 15 to 52 days.

The faecal samples that were frozen in the field were maintained at −20°C until reaching the Endocrine Research Laboratory, University of Pretoria, South Africa. They were then lyophilized using a freeze-dryer (model ALPHA 1–2 LD plus, Christ, Osterode, Germany) for 5 days at −56°C.

### Steroid extraction and analysis

**Figure 2:**
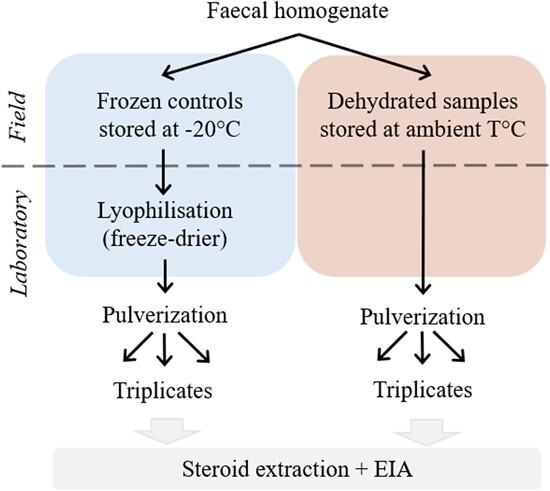
Summary diagram of the experimental process. Each individual faecal sample was homogenized and divided into two sub-samples, one following the gold standard protocol (frozen controls stored at −20°C in the field and then freeze-dried in the laboratory; on the left of the diagram in blue) and the other one following the alternative field-friendly protocol (dehydration and storage at ambient temperature; on the right of the diagram in red).

All samples were pulverized and passed through a mesh sieve to remove undigested material (Ganswindt *et al.,* 2005). For each individual sample and each drying method, we performed three independent steroid extractions using 0.050–0.055 g of faecal powder in 3 ml of 80% ethanol–water (Figure 2). After vortexing for 15 min, the mixture was centrifuged at 1500*g* for 10 min and the supernatant was transferred into sealed microcentrifuge tubes for storage at −20°C until hormone analysis (Ganswindt *et al.,* 2010).

Steroid extracts were analysed using an established 11-oxoaetiocholanolone Enzyme Immunoassay (EIA) detecting fGCMs with a 5β-3α-ol-11-one structure (Möstl *et al.,* 2002; Ganswindt *et al.,* 2003). The sensitivity of the assay was 1.5 nanograms per gram of faecal dry weight (ng/g DW). Intra- and inter-assay coefficients of variation, determined by repeated measurements of low- and high-concentration quality controls were 4.9 and 6.8% as well as 9.8 and 10.4%, respectively. The average standard deviation of triplicates was 33 ng/g DW for the freeze-dried samples and 31 ng/g DW for the dehydrated ones. In addition, the average coefficients of variation of triplicates were 10.0 and 10.8% for the freeze-dried and the dehydrated samples, respectively. Assay procedures followed published protocols (Ganswindt *et al.,* 2002) and were conducted in the Endocrine Research Laboratory, University of Pretoria, South Africa.

### Statistical analysis

We determined whether the individual sample mean fGCM concentrations differed between the two drying and storage procedures using a paired *t* test. We performed a linear regression to estimate the equation line describing the relationship between fGCM concentrations from the two sample drying methods. The fGCM values were normally distributed, so we then performed a Bland–Altman analysis to evaluate the mean difference in fGCM concentration between the two methods and estimate an agreement interval (Bland and Altman, 1986). Finally, we performed a linear regression to investigate the effect of the storage time on the differences in fGCM concentrations between the two drying methods. All statistical analyses were carried out using R version 4.1.2 (R Core Team, 2021).

## Results

**Figure 3:**
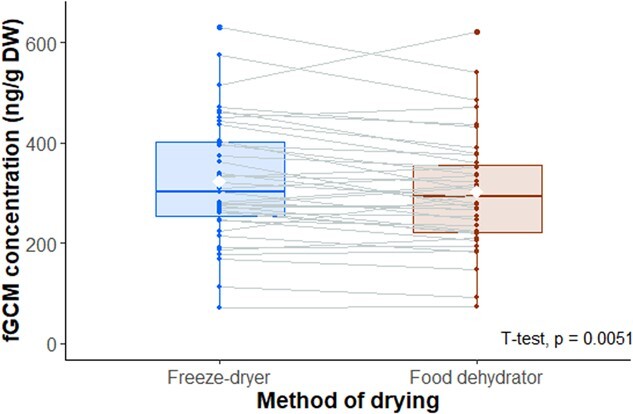
Parallel coordinates plot of fGCM concentrations measured in African elephant faeces (*n* = 40) using two drying and storage protocols. Each point represents the mean of the triplicates. The boxplot on the left (in blue) shows the faecal samples stored at −20°C from collection to laboratory and lyophilized in a freeze-dryer. The boxplot on the right (in red) shows the sub-samples from the same faecal bolus dried in the field in a food dehydrator and stored at ambient temperature (15–25°C). The median, lower and upper quartiles are represented by boxes, the range is represented by vertical lines and the white diamonds represent the means. The paired *t* test shows a significant *P*-value, *P* = 0.0053.

The mean concentration of fGCM measured in the 40 elephants was 332 ± 132 SD ng/g DW when the faeces were stored at −20°C and freeze-dried before analyses, whereas it was 311 ± 127 SD ng/g DW when dehydrated in the field and then maintained at ambient temperature. The loss of fGCM was 6%; however, this difference was statistically significant (t = 2.9506, *P* = 0.0053) (Figure 3).

**Figure 4:**
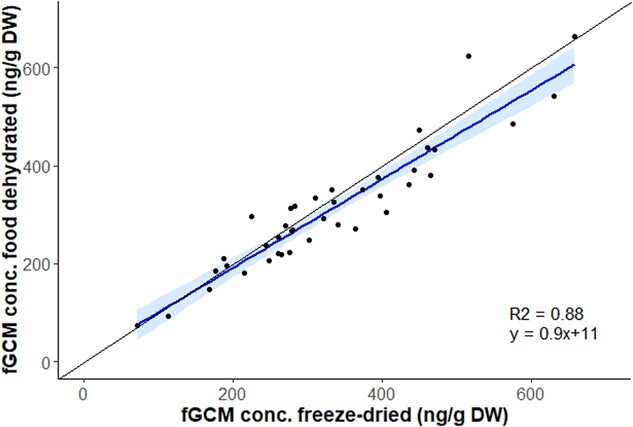
Relationship between fGCM concentrations measured in African elephant faeces (*n* = 40) using two drying and storage protocols. The *x*-axis represents fGCM concentrations obtained with samples stored at −20°C and lyophilized using a freeze-drier; the *y*-axis represents fGCM concentrations obtained with samples dehydrated in the field using a food dehydrator and stored at ambient temperature. The blue solid line represents the linear regression (y = 0.9x + 11, R^2^ = 0.88, *P* < 2.2e-16) and the light blue shade represents the confidence interval (0.95). The black dashed line represents the theoretical straight-line (*y* = *x*).

There was a strong linear relationship between fGCM concentrations measured using the two protocols; the slope of the relationship was close to 1 (0.90) and the coefficient of determination was R^2^ = 0.88, *P* < 2.2e-16 (Figure 4).

**Figure 5:**
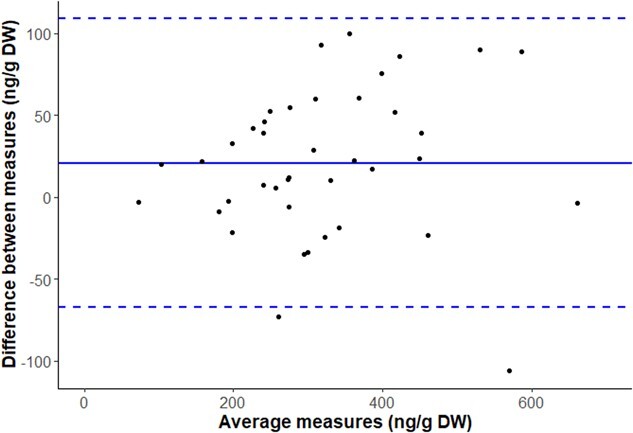
Bland-Altmann plot of the agreement between the two protocols for measuring fGCM concentrations in African elephant faeces (*n* = 40). The *x*-axis represents the average measures of the two protocols, whereas the *y*-axis represents the difference between the two protocols. The solid line represents the mean difference, also called ‘the bias’, and the dashed lines represent the limits of agreement, defined as the mean difference ±1.96 times the standard deviation of the differences. In this study, the mean difference between the two protocols was 21 ng/g DW, with the upper and lower limits of agreement at 110 and −67 ng/g DW, respectively. Most of the data points fall within the limits of agreement, indicating good agreement between the two methods.

The Bland–Altmann plot indicated a mean difference, also called ‘the bias’, between freeze-dried and dehydrated values of 21 ng/g DW, or 6% of difference, and the limits of agreement were between −67 and 110 ng/g DW, within which 95% of the differences between the two protocols fell (Figure 5).

**Figure 6:**
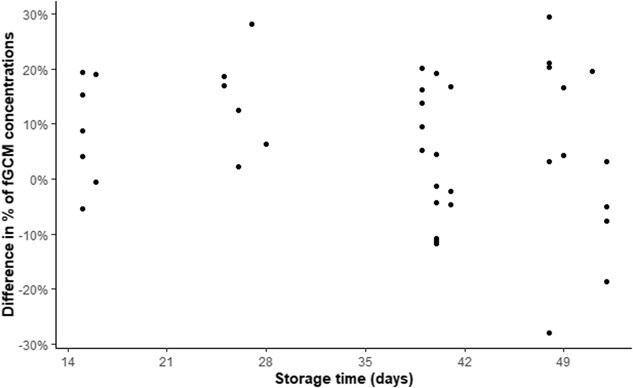
No effect of storage time on the difference between the two measures of fGCM concentrations (stored at −20°C and freeze-dried vs dehydrated in the field and stored at ambient temperature until analysis) in *n* = 40 African elephants (F-statistic = 1.82; *P* = 0.18).

Storage time at ambient temperature of faecal samples, ranging from 15 to 52 days, had no effect on the difference in fGCM concentrations between the two protocols (F-statistic = 1.82; *P* = 0.18) (Figure 6).

## Discussion and Conclusion

Our results demonstrate that the protocol combining food dehydrator and desiccant is suitable for dehydrating African elephant faeces without compromising the fGCM integrity and thus provides a field-friendly alternative to the often logistically challenging process of freezing samples on-site and storing them frozen until analysis. Although the paired *t* test indicates a statistically significant difference in fGCM concentrations between the two protocols (t = 2.9506, *P* = 0.0053), the very predictive regression (R^2^ = 0.88, slope = 0.9, Figure 4) indicates a robust relationship, although with a slight underestimation of fGCM with the food dehydrator protocol. This underestimation is mostly noticeable at relatively higher fGCM levels, i.e. >400 ng/g DW, and this does not contradict the fact that we can clearly generate biologically meaningful results with the food dehydrator approach. The Bland–Altman analysis provided a simple and accurate way to quantify the limits of agreement between two variables (Doğan, 2018), within which 95% of the difference of fGCM values following the field dehydration protocol, compared with the gold standard freezing procedure, fell. This additional analysis enabled us to compare further our method against the gold standard. The small mean difference of 21 ng/g DW, or the ‘bias’, between the two protocols indicates that, on average, the fGCM concentration is slightly underestimated when measured via the food dehydrator (of ~6% on average). With a mean standard deviation of triplicates of ~30 ng/g DW for both protocols, such bias, although statistically significant, is therefore less than the random technical noise associated with the fGCM measurement. This small bias value and the reasonable limits of agreement ranging from −67 to 110 ng/g DW again indicate that the use of dehydration protocol in the field should not affect our interpretation of revealed fGCM concentrations and is unlikely to influence elephant management decisions differently. Nevertheless, the slight overall underestimation of fGCMs with our protocol should be taken into consideration, particularly if comparisons are made with data obtained using another sample conservation protocol. We also checked that the dehydration protocol in the field did not lead to repeatability issues; the mean standard deviation of the triplicates was very similar with both methods, i.e. 31 ng/g DW for the field dehydrated samples versus 33 ng/g DW with the standard freezing procedure, as well as the mean coefficient of variation, i.e. 10.0 and 10.8%, respectively, showing a similar dispersion around the mean.

Finally, we ensured that the fGCMs were stable when the faeces dehydrated in the field were stored at ambient temperature, a necessary condition to secure storage and transport of the samples while minimizing logistical constraints. We found no significant effect of storage time at ambient temperature on the differences between the two protocols up to 52 days (F-statistic = 1.82; *P* = 0.18). Although there was no observable trend of increasing or decreasing fGCMs over time, it cannot be ruled out that analyses conducted on a larger sample size would not have slightly altered our conclusions. We also cannot exclude that the differences in fGCM concentrations may be affected over a longer storage time, as observed with other faeces storage methods (Hunt and Wasser, 2003; Pappano *et al.,* 2010). However, the long-term stability of fGCMs should be secured provided that the bacterial activity is kept suppressed (Bokkenheuser and Winter, 1980; Möstl *et al.,* 1999; Lexen *et al.,* 2008), i.e. as long as the samples are kept free of moisture (Möstl *et al.,* 2005). It is thus reasonable to expect that storage of faeces at ambient temperature should be possible for a longer period provided the samples are kept dry (i.e. stored in sealed tubes or maintained in a dry atmosphere). Further experiments are needed to confirm whether there is a limit to the storage time of faeces dehydrated following our protocol.

The use of a food dehydrator and desiccant provides an easy and cost-effective alternative to keeping the samples frozen, preferable when a limited power supply is available or in a remote study area, which may involve complicated transport of samples. Compared with other methods where samples are also dried directly in the field (e.g. sunlight exposure), this alternative method also suppresses any reliance on weather conditions and protects the samples from insects and birds. The drying method using silica beads in contact with faeces has not proved effective in elephants for preserving samples at ambient temperature (Hunt and Wasser, 2003), and therefore does not make it possible to dispense with a freezer in the field. However, we deduced that the addition of silica crystals as a desiccant in the food dehydrator would indeed hasten and optimize the drying process. These aspects indicate that the use of a food dehydrator with desiccant could be an excellent tool to improve and develop the monitoring of African elephant physiological stress in areas where such monitoring programs were not implemented before for logistical reasons.

In a context of growing interest in African elephant welfare in South Africa (South African Department of Forestry, Fisheries and the Environment (DFFE), 2020), the need to consider such physiological metrics in conservation management decision-making is becoming crucial. In addition, the application of a clinical analytical method, such as the Bland–Altman analysis, adds a stone to the edifice of a transdisciplinary approach to conservation and provides an easy tool to researchers to test new field-friendly methods of interest for conservationists. Future research should thus focus on the efficacy of this alternative to freeze-drying for other species and different steroid classes.

## Funding

This work was supported by the French doctoral school Evolution, Ecosystèmes, Microbiologie, Modélisation (E2M2), Claude Bernard Lyon 1 University and the French National Centre for Scientific Research (CNRS) through allocations to the International Research Laboratory REHABS.

## Competing interests

The authors have no conflicts of interest to declare.

## Data availability

The data underlying this article will be available from the corresponding author, L.L., on reasonable request.

## Author contributions

L.L.: Methodology, data collection, analysis, visualization, writing (original draft). C.G., H.F.: Conceptualization, methodology, supervision, writing (reviews). A.G.: Conceptualization, methodology, writing (reviews). B.R.: Conceptualization, methodology, visualization, supervision, writing (reviews). All authors discussed the results and contributed to the final manuscript.

## References

[ref1] Bland JM , Altman D (1986) Statistical methods for assessing agreement between two methods of clinical measurement. The lancet 327: 307–310. 10.1016/S0140-6736(86)90837-8. 2868172

[ref2] Bokkenheuser VD , Winter J (1980) Biotransformation of steroid hormones by gut bacteria. Am J Clin Nutr 33: 2502–2506. 10.1093/ajcn/33.11.2502. 7001886

[ref3] Busch DS , Hayward LS (2009) Stress in a conservation context: a discussion of glucocorticoid actions and how levels change with conservation-relevant variables. Biol Conserv 142: 2844–2853. 10.1016/j.biocon.2009.08.013.

[ref4] Cannon WB (1929) Organization for physiological homeostasis. Physiol Rev 9: 399–431. 10.1152/physrev.1929.9.3.399.

[ref5] Carbillet J , Palme R, Maublanc M-L, Cebe N, Gilot-Fromont E, Verheyden H, Rey B (2023) Instability of fecal glucocorticoid metabolites at 4°C: time to freeze matters. J Exp Zool Pt A 339: 625–632. 10.1002/jez.2704. 37058280

[ref6] Dantzer B , Fletcher QE, Boonstra R, Sheriff MJ (2014) Measures of physiological stress: a transparent or opaque window into the status, management and conservation of species? Conservation Physiology 2: cou023. 10.1093/conphys/cou023. 27293644 PMC4732472

[ref7] Doğan NÖ (2018) Bland-Altman analysis: a paradigm to understand correlation and agreement. Turkish Journal of Emergency Medicine 18: 139–141. 10.1016/j.tjem.2018.09.001. 30533555 PMC6261099

[ref8] Edwards KL , McArthur HM, Liddicoat T, Walker SL (2014) A practical field extraction method for non-invasive monitoring of hormone activity in the black rhinoceros. Conservation physiology 2: cot037. 10.1093/conphys/cot037. 27293621 PMC4732489

[ref9] Foley CAH , Papageorge S, Wasser SK (2001) Noninvasive stress and reproductive measures of social and ecological pressures in free-ranging African elephants. Conserv Biol 15: 1134–1142. 10.1046/j.1523-1739.2001.0150041134.x.

[ref10] Fowler KJ , Santymire RM (2022) A novel field method for preserving African lion (Panthera leo) fecal samples for noninvasive hormone metabolite analysis. Methods X 9: 101881. 10.1016/j.mex.2022.101881 PMC9641247 36385914

[ref11] Ganswindt A , Brown JL, Freeman EW, Kouba AJ, Penfold LM, Santymire RM, Vick MM, Wielebnowski N, Willis EL, Milnes MR (2012) International Society for Wildlife Endocrinology: the future of endocrine measures for reproductive science, animal welfare and conservation biology. Biol Lett 8: 695–697. 10.1098/rsbl.2011.1181. 22219389 PMC3440958

[ref12] Ganswindt A , Heistermann M, Borragan S, Hodges JK (2002) Assessment of testicular endocrine function in captive African elephants by measurement of urinary and fecal androgens. Zoo Biol 21: 27–36. 10.1002/zoo.10034.

[ref13] Ganswindt A , Heistermann M, Hodges K (2005) Physical, physiological, and behavioral correlates of musth in captive African elephants (Loxodonta africana). Physiol Biochem Zool 78: 505–514. 10.1086/430237. 15957105

[ref14] Ganswindt A , Muenscher S, Henley M, Henley S, Heistermann M, Palme R, Thompson P, Bertschinger H (2010) Endocrine correlates of musth and the impact of ecological and social factors in free-ranging African elephants (Loxodonta africana). Horm Behav 57: 506–514. 10.1016/j.yhbeh.2010.02.009. 20188104

[ref15] Ganswindt A , Palme R, Heistermann M, Borragan S, Hodges JK (2003) Non-invasive assessment of adrenocortical function in the male African elephant (Loxodonta africana) and its relation to musth. Gen Comp Endocrinol 134: 156–166. 10.1016/S0016-6480(03)00251-X. 14511986

[ref16] Garai ME , Roos T, Eggeling T, Ganswindt A, Pretorius Y, Henley M (2022) Developing welfare parameters for African elephants (Loxodonta africana) in fenced reserves in South Africa. PloS One 17: e0264931. 10.1371/journal.pone.0264931. 35324916 PMC8947097

[ref17] Gholib G , Agil M, Supriatna I, Purwantara B, Heistermann M, Engelhardt A (2017) Repeated freeze-thaw cycles but not short-term storage of fecal extracts at ambient temperature influence the stability of steroid metabolite levels in crested macaques. J Ked Hewan 11: 78–85. 10.21157/j.ked.hewan.v11i2.6830.

[ref18] Gholib G , Heistermann M, Agil M, Supriatna I, Purwantara B, Nugraha TP, Engelhardt A (2018) Comparison of fecal preservation and extraction methods for steroid hormone metabolite analysis in wild crested macaques. Primates 59: 281–292. 10.1007/s10329-018-0653-z. 29429140

[ref19] Gholib G , Nugraha TP, Agil M, Supriatna I, Purwantara B, Engelhardt A (2014) Faecal glucocorticoid measurement as indicator stress in wild crested macaques (Macaca nigra): the importance of validation and sample processing techniques. In Presented at the Proceeding Science and Engineering, The 4th Annual International Conference Syiah Kuala University. Banda Aceh, Indonesia.

[ref20] Grotto CE , Wolf T, Berkeley E, Lee S, Ganswindt A (2020) Physiological measure of animal welfare in relation to semi-captive African elephant (Loxodonta africana) interaction programs. African Zoology 55: 245–249. 10.1080/15627020.2020.1776635.

[ref21] Hunt KE , Wasser SK (2003) Effect of long-term preservation methods on fecal glucocorticoid concentrations of grizzly bear and African elephant. Physiol Biochem Zool 76: 918–928. 10.1086/380209. 14988807

[ref22] Khan MZ , Altmann J, Isani SS, Yu J (2002) A matter of time: evaluating the storage of fecal samples for steroid analysis. Gen Comp Endocrinol 128: 57–64. 10.1016/S0016-6480(02)00063-1. 12270788

[ref23] Krueger K , Marr I, Dobler A, Palme R (2019) Preservation of fecal glucocorticoid metabolites and immunoglobulin A through silica gel drying for field studies in horses. Conservation Physiology 7: coz065. 10.1093/conphys/coz065. 31687143 PMC6821355

[ref24] Kumar V , Pradheeps M, Kokkiligadda A, Niyogi R, Umapathy G (2019) Non-invasive assessment of physiological stress in captive Asian elephants. Animals 9: 553. 10.3390/ani9080553. 31416158 PMC6720305

[ref25] Lexen E , El-Bahr SM, Sommerfeld-Stur I, Palme R, Mostl E (2008) Monitoring the adrenocortical response to disturbances in sheep by measuring glucocorticoid metabolites in the faeces. Wiener Tierarztliche Monatsschrift 95: 64.

[ref26] Madliger CL , Love OP, Hultine KR, Cooke SJ (2018) The conservation physiology toolbox: status and opportunities. Conservation Physiology 6: coy029. 10.1093/conphys/coy029. 29942517 PMC6007632

[ref27] Millspaugh JJ , Burke T, Dyk GV, Slotow R, Washburn BE, Woods RJ (2007) Stress response of working African elephants to transportation and safari adventures. The Journal of Wildlife Management 71: 1257–1260. 10.2193/2006-015.

[ref28] Möstl E , Maggs JL, Schrötter G, Besenfelder U, Palme R (2002) Measurement of cortisol metabolites in faeces of ruminants. Vet Res Commun 26: 127–139. 10.1023/A:1014095618125. 11922482

[ref29] Möstl E , Messmann S, Bagu E, Robia C, Palme R (1999) Measurement of glucocorticoid metabolite concentrations in faeces of domestic livestock. Journal of Veterinary Medicine Series A: Physiology Pathology Clinical Medicine 46: 621–631. 10.1046/j.1439-0442.1999.00256.x. 10638300

[ref30] Möstl E , Rettenbacher S, Palme R (2005) Measurement of corticosterone metabolites in birds’ droppings: an analytical approach. Ann N Y Acad Sci 1046: 17–34. 10.1196/annals.1343.004. 16055841

[ref31] Mumby HS , Mar KU, Thitaram C, Courtiol A, Towiboon P, Min-Oo Z, Htut-Aung Y, Brown JL, Lummaa V (2015). Stress and body condition are associated with climate and demography in Asian elephants., Conservation Physiology 3: cov030. 10.1093/conphys/cov030. 27293715 PMC4778474

[ref32] Nugraha TP , Heistermann M, Agil M, Purwantara B, Supriatna I, Gholib G, van Schaik CP, Weingrill T (2017) Validation of a field-friendly extraction and storage method to monitor fecal steroid metabolites in wild orangutans. Primates 58: 285–294. 10.1007/s10329-016-0583-6. 27771831

[ref33] Palme R (2019) Non-invasive measurement of glucocorticoids: advances and problems. Physiol Behav 199: 229–243. 10.1016/j.physbeh.2018.11.021. 30468744

[ref34] Pappano DJ , Roberts EK, Beehner JC (2010) Testing extraction and storage parameters for a fecal hormone method. Am J Primatol 72: 934–941. 10.1002/ajp.20859. 20623500

[ref35] Pokharel SS , Seshagiri PB, Sukumar R (2017) Assessment of season-dependent body condition scores in relation to faecal glucocorticoid metabolites in free-ranging Asian elephants. Conservation Physiology 5: cox039. 10.1093/conphys/cox039. 28721215 PMC5508666

[ref36] Pokharel SS , Singh B, Seshagiri PB, Sukumar R (2018) Lower levels of glucocorticoids in crop-raiders: diet quality as a potential ‘pacifier’ against stress in free-ranging Asian elephants in a human-production habitat. Animal Conservation 22: 177–188. 10.1111/acv.12450.

[ref37] Postiglione G , Accorsi PA, Ganswindt A, Crossey B (2022) A field-friendly alternative to freeze-drying faeces for glucocorticoid metabolite analyses of African wild dogs (Lycaon pictus). Methods X 9: 101623. 10.1016/j.mex.2022.101623. PMC8790624 35111576

[ref38] R Core Team (2021) R: A language and environment for statistical computing. R Foundation for Statistical Computing, Vienna, Austria, https://www.R-project.org/

[ref39] Sheriff MJ , Dantzer B, Delehanty B, Palme R, Boonstra R (2011) Measuring stress in wildlife: techniques for quantifying glucocorticoids. Oecologia 166: 869–887. 10.1007/s00442-011-1943-y. 21344254

[ref40] Shutt K , Setchell JM, Heistermann M (2012) Non-invasive monitoring of physiological stress in the Western lowland gorilla (Gorilla gorilla gorilla): validation of a fecal glucocorticoid assay and methods for practical application in the field. Gen Comp Endocrinol 179: 167–177. 10.1016/j.ygcen.2012.08.008. 22926327

[ref41] South African Department of Forestry, Fisheries and the Environment (DFFE) (2020) The High-Level Panel of Experts for the Review of Policies, Legislation and Practices on Matters of Elephant, Lion, Leopard and Rhinoceros Management, Breeding, Hunting, Trade and Handling. South Africa, p. 582. https://www.environment.gov.za/sites/default/files/reports/2020-12-22_high-levelpanel_report.pdf.

[ref41a] Szott ID , Pretorius Y, Ganswindt A, Koyama NF (2020) Physiological stress response of African elephants to wildlife tourism in Madikwe game reserve, South Africa. Wildl Res 47: 34. 10.1071/WR19045.

[ref43] Viljoen JJ , Ganswindt A, Palme R, Reynecke HC, Du Toit JT, Langbauer WR Jr (2008) Measurement of concentrations of faecal glucocorticoid metabolites in free-ranging African elephants within the Kruger National Park. Koedoe 50: 18–21. 10.4102/koedoe.v50i1.129.

[ref44] Webber JT , Henley MD, Pretorius Y, Somers MJ, Ganswindt A (2018) Changes in African elephant (Loxodonta africana) faecal steroid concentrations post-defaecation. Bothalia - African Biodiversity &amp; Conservation 48: 1–8.

[ref45] Wingfield JC , Maney DL, Breuner CW, Jacobs JD, Lynn S, Ramenofsky M, Richardson RD (1998) Ecological bases of hormone-behavior interactions: the emergency life history stage. Am Zool 38: 191–206. 10.1093/icb/38.1.191.

